# A Long-Lasting Textile-Based Anatomically Realistic Head Phantom for Validation of EEG Electrodes

**DOI:** 10.3390/s21144658

**Published:** 2021-07-07

**Authors:** Granch Berhe Tseghai, Benny Malengier, Kinde Anlay Fante, Lieva Van Langenhove

**Affiliations:** 1 Department of Materials, Textiles and Chemical Engineering, Ghent University, 9000 Gent, Belgium; Benny.Malengier@UGent.be (B.M.); Lieva.VanLangenhove@UGent.be (L.V.L.); 2 Jimma Institute of Technology, Jimma University, Jimma, Ethiopia; kinde.anlay@ju.edu.et

**Keywords:** e-textile, head phantom, electroencephalography, conductive material

## Abstract

During the development of new electroencephalography electrodes, it is important to surpass the validation process. However, maintaining the human mind in a constant state is impossible which in turn makes the validation process very difficult. Besides, it is also extremely difficult to identify noise and signals as the input signals are not known. For that reason, many researchers have developed head phantoms predominantly from ballistic gelatin. Gelatin-based material can be used in phantom applications, but unfortunately, this type of phantom has a short lifespan and is relatively heavyweight. Therefore, this article explores a long-lasting and lightweight (−91.17%) textile-based anatomically realistic head phantom that provides comparable functional performance to a gelatin-based head phantom. The result proved that the textile-based head phantom can accurately mimic body-electrode frequency responses which make it suitable for the controlled validation of new electrodes. The signal-to-noise ratio (SNR) of the textile-based head phantom was found to be significantly better than the ballistic gelatin-based head providing a 15.95 dB ± 1.666 (±10.45%) SNR at a 95% confidence interval.

## 1. Introduction

Measuring the electrical activity in the brain, heart, muscles, etc., using electrodes to know the health condition of humans and/or animals is a common clinical practice. However, such electrodes have to be validated prior to being employed in clinical practices. For instance, PEDOT/PSS-based and silver-based electrocardiography (ECG) electrodes have been developed [1] to measure heart activity but a scientific validation was not performed as part of that research as ECG signals were different from person to person and even for the same person over time. Electroencephalography (EEG) measurements to monitor brain activity are much more variable with changes over seconds.

**Figure 1:**
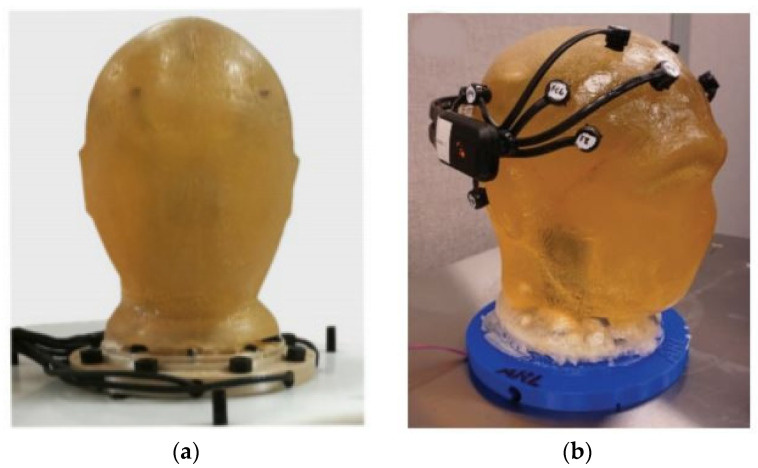
Examples of gelatin-based phantoms: (**a**) from [7]; (**b**) from [8].

For the validation of EEG electrodes, it is, therefore, required to develop head phantoms as maintaining a constant brain activity is hardly possible. Hence, it is required to conduct a test in an environment as realistic as possible with a known ground truth of source location and brain activity. This can be performed via digital phantoms by modeling the propagation of the signal originating within the brain to the electrodes [2]. However, the studies via digital head phantom are hardly suited to mimic motion artifacts of a realistic EEG, electromagnetic interference noise generated by the power lines, and high power electronic equipment [3]. For that reason, many researchers have developed head phantoms predominantly from ballistic gelatin [4,5,6,7,8]. Gelatin-based materials are a good material to be used in phantom applications, but unfortunately, this type of phantom has a short life span [9] and is too heavyweight. Examples of gelatin-based head phantoms are shown in Figure 1.

Recently, Tsizin et al. developed a realistic head phantom mimicking the electromagnetic properties of the head where the internal volume of a human skull was filled with a conductive gel [10]. However, the lifetime of the phantom was only about a month. Other EEG head phantoms [11,12] prepared by casting were also introduced but still, the casting process is complicated, the phantoms are heavy and expensive. Therefore, developing a simple lightweight and long-lasting textile-based head phantom would be an important improvement.

The emergence of electrically conductive textiles led textile materials to a versatile application in the electronic and medical industries [13]. Electrically conductive textiles can be developed by different techniques and in different forms [14]. Moreover, the electrical and physical properties of the textile substrate can be easily controlled, and the required extent of stretchability, flexibility, and conductivity can be imparted by regulating the substrate, textile construction, and application of the conductive component. Therefore, this work explores the use of e-textiles for a head phantom.

## 2. Materials and Methods

### 2.1. Head Phantoms Construction

**Figure 2:**
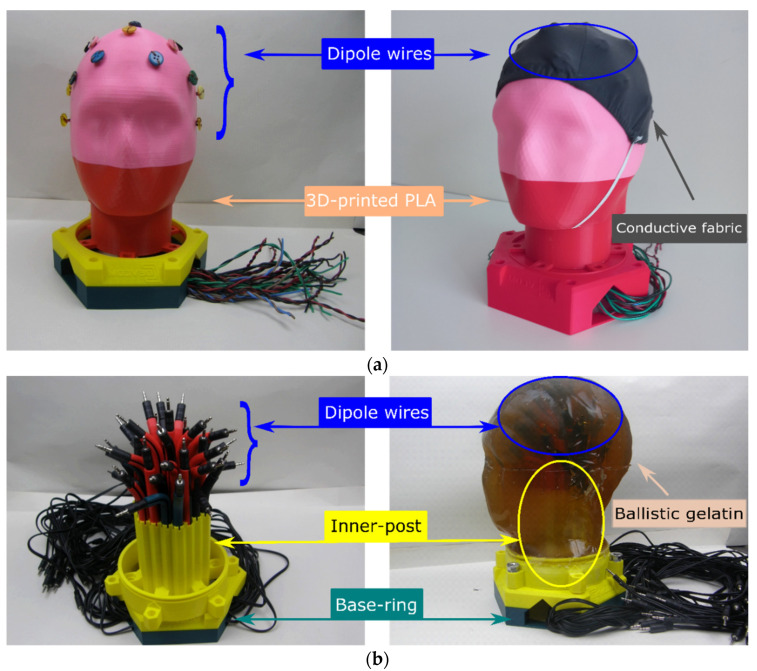
Head Phantom: (**a**) textile-based; (**b**) ballistic gelatin-based.

A textile-based head phantom was constructed by placing a bi-directional stretchy nylon/spandex (18:7) EeonTex conductive stretchable fabric (obtained from MANDU, Finland) over an anatomically realistic 3D-print polylactic acid (PLA) skull. The conductive fabric has a surface resistivity that can be custom-tuned for specific requirements in the range of 10^4^ to 10^7^ Ω/square. To mimic the neurons, twenty (20) 3.5 mm stereo male–male dipole wires were installed underneath the conductive fabric per the 10–20 EEG placement system as shown in Figure 2a. Side to side, a gelatin-based head phantom was also constructed from 900 g gelatin, 40.5 g table salt, and 4.5 L demineralized water according to [15], for comparison. Thirty-seven (37) dipole wires were installed inside the ballistic gelatin as shown in Figure 2b. The skull, base-ring, inner-post, and guiding wires have been constructed from PLA using an FDM 3D printer at Ingegno Maker Space (Drongen, Belgium). The photographic images of the constructed textile and gelatin-based head phantoms and their components are shown in Figure 2a,b, respectively.

### 2.2. Head Phantom Validation

**Figure 3:**
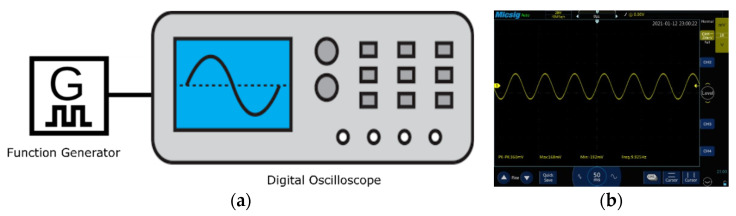
Synthetic sine wave generation: (**a**) wave generation setup using a function generator and digital oscilloscope; (**b**) the photographic image of the generated synthetic sine wave.

To validate the head phantoms, a synthetic sine wave (360 mV peak to peak voltage, 168 mV maximum voltage, −192 mV minimum voltage, 9.925 Hz frequency) was generated using a function generator DDS Function Signal Generator and recorded with a handheld tablet digital oscilloscope (Micsig TO1104). This was then injected into the head phantoms as shown in Figure 3. To impersonate events, the electroencephalography (EEG) phantom signal parameters were set in the alpha wave range and the amplitude was varied with the function generator to mimic a neurological event.

**Figure 4:**
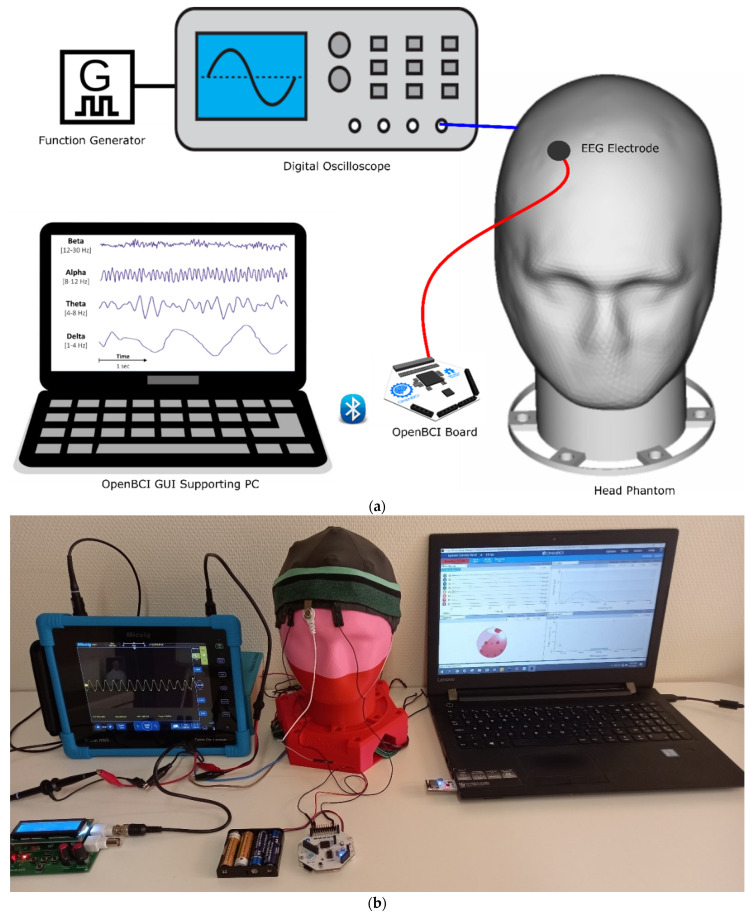
Measurement-setup: (**a**) schematic illustration; (**b**) actual.

The head phantom replaces a real human head, and EEG electrodes can be attached as one would do on a human. In this test, the generated EEG wave was measured on both types of head phantoms using an active reusable snap Ag/AgCl dry electrode connected to a Cyton biosensing Board (8-channels) of OpenBCI according to the setup in Figure 4.

### 2.3. Phantom-to-Electrode Impedance

The head phantom-to-electrode impedance was measured using a three-electrode configuration (reference, counter, and active electrodes), also with the Cyton Biosensing (OpenBCI) board and reusable snap Ag/AgCl dry EEG electrodes to study the difference between the ballistic gelatin and textile-based head phantoms. The system was adopted from OpenBCI and was suggested to measure skin-to-electrode impedance as the OpenBCI Cython board has an installed ADS1299 to measure impedance. A 5 kΩ resistor is built into the OpenBCI board in series to each electrode and has to be taken into account. The ADS1299 has a feature called “Lead Off Detection” that can do the impedance measurement by injecting a known current into each electrode. A 6 nA current is forced into the electrode line by a current source built into the ADS1299 [16], regardless of how much resistance or impedance there is between the current source and the ground (within reason). Hence, a 6 nA current will be present through the electrode to the ground during this test. For this work, only the head phantoms were used, no humans. Therefore, the impedance was calculated using Equation (1), where the current is 6 × 10^−9^ A. Then, the phantom-to-electrode impedance was analytically calculated.

(1) $$Average\;Impedance(\Omega )=\frac{Average\;Voltage\left(V\right)}{Current\left(I\right)}$$

However, the average voltages collected during the test are in root mean square voltages (*Vrms*). Thus, the *average voltage* was calculated using Equation (2).

(2) $$Average\;Voltage=\frac{Vrms\times 2\sqrt{2}}{\pi }=\frac{Vrms}{1.1107}$$

Finally, the average impedance here is the series resistance of the head phantom-to-electrode interface and the 5 kΩ resistor built into the OpenBCI board. So, to obtain the actual impedance of just the phantom-to-electrode interface, one needs to subtract 5 kΩ from the average impedance as in Equation (3).

(3) Actual Average Impedance(Ω)=Average Impedance(Ω)−5000

### 2.4. Signal Analysis

The quality of signals collected was mathematically analyzed in terms of Signal-to-Noise Ratio (SNR) using Equation (4). The peak-to-peak voltage signal is the synthetic peak-to-peak voltage injected from the digital oscilloscope to the head phantom and the peak-to-peak voltage signal is the difference between the injected and collected back peak-to-peak voltage signal.

(4) $$SNR\left(dB\right)=10log\left(\frac{Peak\;to\;Peak\;Voltage\;Signal}{Peak\;to\;Peak\;Voltage\;Noise}\right)$$

The event-related spectral perturbation (ERSP) and inter-trial coherence (ITC) time-frequency measurements were then processed and analyzed via EEGLAB software that is treated as in Equation (5) according to spectral and coherence estimates on EEG recordings [17]. ITC is computed from single-trial EEG to reflect the temporal and spectral synchronization within EEG, explaining the extent to which underlying phase-locking occurs [17].

(5) $$ITC\left(f,t\right)=\frac{1}{n}\overset{n}{\underset{k=1}{\sum }}\frac{F_{k}\left(f,t\right)}{F_{k}\left(f,t\right)\vee }$$

where *F*, *t* and *n* denote frequency, time and amount of data, respectively.

## 3. Results and Discussion

The new textile-based head phantom has a much lighter weight than the gelatin-based i.e., 0.5 and 6 kg, respectively. Therefore, the weight reduction is 91.67% which makes it more suitable for handling and moving from place to place. In addition, it is not delicate like the ballistic gelatin-based, where the shape of ballistic gelatin could be distorted and decays fast even when kept in a refrigerator. In our case, the gelatin-based head phantom begun decaying after a week of its construction which may also depend on the weather where it is placed during testing. In contrast, the textile-based head phantom does not decay at all.

### 3.1. Phantom-to-Electrode Impedance

The results in Table 1 indicate that the impedance of the textile-based head phantom is significantly lower with an f-ratio value of 2123.35 and a *p*-value of <0.001 at a 95% confidence interval according to one-way ANOVA. It is 1863 Ω for the textile-based head phantom and 2297 Ω, so they are in the same operating range. For comparison, a skin-to-electrode impedance measurement was performed on a human with the OpenBCI board and was found to be in the range of 3239.55 Ω to 1991.09 Ω, which is in the same range as the textile-based head phantom. The lower impedance means the long-lasting and lightweight textile-based head phantom can collect somewhat better-quality signals than the gelatin-based head phantom which would make it preferable for validating EEG electrodes in particular and other bio-potential electrodes in general. The head phantom can also potentially be used during modeling and simulation work related to brain neurological activities.

### 3.2. Electroencephalogram (EEG) Signal

EEG is a term for the electrical signals of the brain [18] and was introduced by Hans Berger in 1929 [19]. Electrodes located outside (noninvasive brain-computer interface) of our brain, i.e., on the human scalp, are used to measure EEG. The frequency is the most common method for classifying EEG waveforms, to the point that EEG waves are denoted using Greek numerals based on their frequency spectrum. Delta (0.5 to 4 Hz), theta (4 to 7 Hz), alpha (8 to 12 Hz), sigma (12 to 16 Hz), and beta are the most widely studied waveforms (13 to 30 Hz).

**Figure 5:**
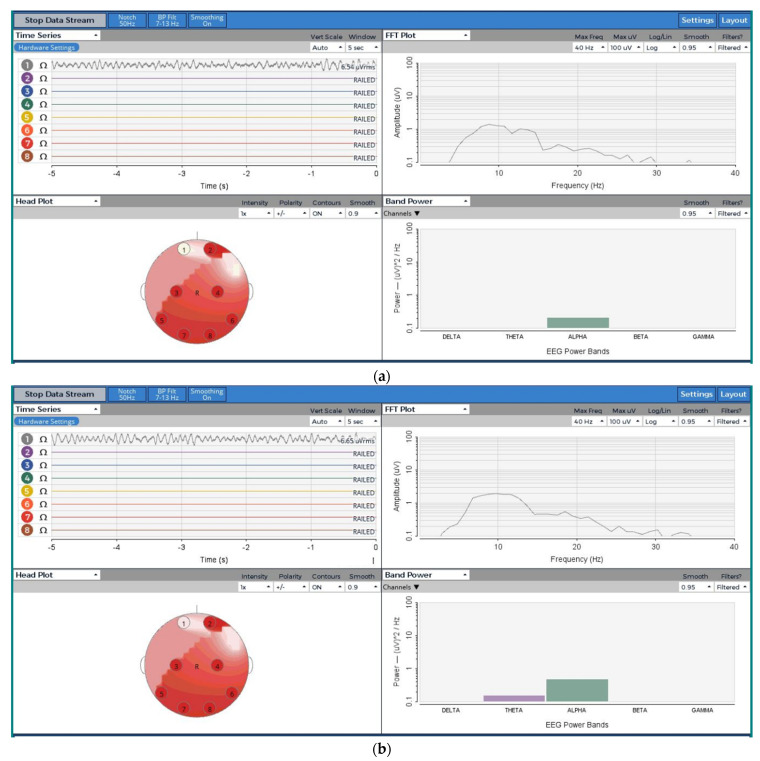
EEG signal from OpenBCI board: (**a**) textile-based head phantom; (**b**) gelatin-based head phantom.

The textile-based head phantom allowed for the injection of well-defined synthetic waves using a digital oscilloscope, and collection of the EEG waveform using an OpenBCI board, strongly similar and matching to the gelatin-based. The EEG wave collected from the textile-based head phantom predominantly lays in the alpha band, the same as the injected sine wave. Whereas, from the ballistic gelatin, a very small theta band was observed where an injected band power was generated. From the EEG band powers in Figure 5, the noise in the textile-based head phantom was less, however, statistically, the root-mean-square voltages (Vrms) from the time series in Figure 5 in both phantoms were not significantly different at 95% of confidence interval according to one-way ANOVA. The frequency vs. FFT (Fast Fourier Transform) plot showed that the amplitude and frequencies were strongly similar and in the same range, in addition, the head plot was also quite similar. Therefore, this textile-based head phantom can potentially replace the gelatin-based head for validating EEG electrodes.

### 3.3. SNR Analysis

From Table 2, the SNR of the textile-based head phantom was found to be significantly better than the gelatin-based one. The marginal error was 15.95 dB ± 1.666 (±10.45%) with a 95% confidence interval. Therefore, textile-based head phantoms are preferable.

### 3.4. Inter-Trial Coherence (ITC) and Event-Related Spectral Perturbation (ERSP)

**Figure 6:**
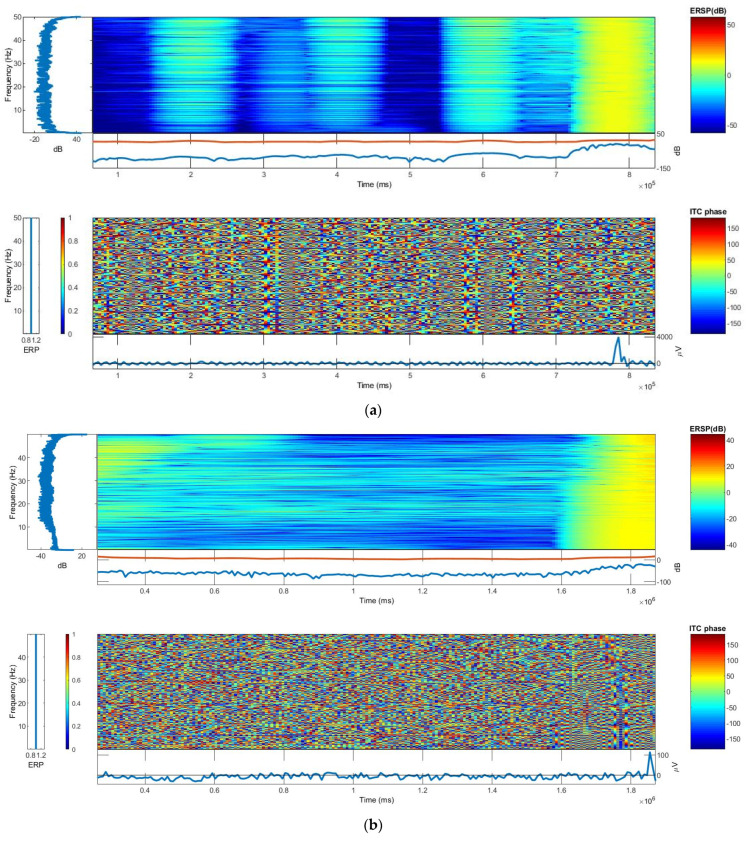
ITC and ERSP: (**a**) gelatin-based head phantom; (**b**) textile-based head phantom.

The frequency and time ranges are plotted on the y-axis and x-axis, respectively, and a color scale is used, with green representing non-significant ITC and red representing significant ITC at a 99% confidence interval. The averaged ERP response for that person (in blue) is plotted beneath each ITC plot. The ERP response amplitude scale for both phantoms is somewhat close in this study. From EEGLAB software analysis, the log power spectral density for both the CDE and TE was ~90 dB. However, the distribution of spectral powers was more uniform in the textile-based main phantom. The ITC and ERP plots of the textile-based and gelatin-based head phantoms are shown in Figure 6.

## 4. Conclusions

Keeping the human brain constant is hardly possible. Therefore, anatomically realistic head phantoms should be used to validate bio-potential electrodes such as for an electroencephalogram (EEG). In this work, we explored a long-lasting and lightweight head phantom that allows synthetic wave injection and measuring at a performance similar to the commonly used ballistic gelatin-based head phantoms. It was found to perform similarly, and for some users even better than the gelatin-based one. While the textile-based phantom was designed for EEG, it can also be adapted to electrocardiogram, electromyogram, electrooculogram, and other related studies as well.

## Figures and Tables

**Table 1:** Head phantom to electrode impedance.

Test	Time Counter (s)	Textile-Based Head Phantom			Gelatin-Based Head Phantom		
		V_raw_	Z_raw_	Z_act_	V_raw_	Z_raw_	Zact
1	30	41.18	6863	1863	43.93	7321	2321
2	60	43.81	7301	2301	43.98	7330	2330
3	90	42.66	7110	2110	44.54	7423	2423
4	120	42.92	7153	2153	43.87	7311	2311
5	150	43.13	7188	2188	42.49	7081	2081
6	180	42.44	7073	2073	44.12	7353	2353
7	210	41.33	6888	1888	43.72	7286	2286
8	240	42.72	7120	2120	43.63	7271	2271
**Mean**		**41.18**	**6863**	**1863**	**43.79**	**7297**	**2297**

V_raw_ = Raw Average Voltage (µV), Z_avg_ = Raw Average Impedance (Ω), Z_act_ = Actual Average Impedance (Ω).

**Table 2:** Injected wave, acquired signal, and SNR of the head phantoms.

	Wave	V Max (mV)	V Min (mV)	V Pk-Pk (mV)	SNR (dB)
Function generator	Synthetic Signal	168.00	−192.00	360.00	
Gelatin-based head phantom	Signal	164.92	−184.30	349.22	15.1
	Noise	3.08	−7.701	10.78	
	Signal/Noise	0.054	0.024	0.032	
Textile-based head phantom	Signal	166.83	−185.80	352.63	16.8
	Noise	1.17	−6.20	7.37	
	Signal/Noise	0.142	0.03	47.84	
